# A multiplexed microflow LC–MS/MS-PRM assay for serologic quantification of IgG N- and HPX O- glycoforms in liver fibrosis

**DOI:** 10.1038/s41598-023-27382-0

**Published:** 2023-01-12

**Authors:** Aswini Panigrahi, Lihua Zhang, Julius Benicky, Miloslav Sanda, Jaeil Ahn, Radoslav Goldman

**Affiliations:** 1 Department of Oncology, Lombardi Comprehensive Cancer Center, Georgetown University, Washington, DC 20057 USA; 2 Clinical and Translational Glycoscience Research Center, Georgetown University Medical Center, Georgetown University, Washington, DC 20057 USA; 3 Max-Planck-Institut fuer Herz-und Lungenforschung, Ludwigstrasse 43, 61231 Bad Nauheim, Germany; 4 Department of Biostatistics, Bioinformatics and Biomathematics, Georgetown University, Washington, DC 20057 USA; 5 Department of Biochemistry and Molecular and Cellular Biology, Georgetown University, Washington, DC 20057 USA

**Keywords:** Glycobiology, Analytical biochemistry

## Abstract

Targeted quantification of glycoproteins has not reached its full potential because of limitations of the existing analytical workflows. In this study, we introduce a targeted microflow LC–MS/MS-PRM method for the quantification of multiple glycopeptides in unfractionated serum samples. The entire preparation of 16 samples in a batch is completed within 3 h, and the LC–MS quantification of all the glycoforms in a sample is completed in 15 min in triplicate, including online capture and desalting. We demonstrate applicability of the workflow on a multiplexed quantification of eight N-glycoforms of immunoglobulin G (IgG) together with two O-glycoforms of hemopexin (HPX). We applied the assay to a serologic study of fibrotic liver disease in patients of HCV etiology. The results document that specific IgG- and HPX-glycoforms detect efficiently fibrotic disease of different degree, and suggest that the LC–MS/MS-PRM assays may provide rapid and reproducible biomarker assay targeting simultaneously the N- and O-glycoforms of the peptides. We propose that such high throughput multiplexed methods may advance the clinical use of the LC–MS/MS assays.

## Introduction

Hepatocellular carcinoma (HCC) is a major human health burden accounting for approximately 90% of primary liver cancer, it is the 3rd most common cause of cancer-related death worldwide, and the global death rate is projected to reach one million by 2030^1–3^. A large majority of these cases are associated with hepatitis B and C infections, and association with nonalcoholic fatty liver disease (NAFLD) and nonalco-holic steatohepatitis (NASH) is increasing. Changes in glycosylation in serum proteins are often associated with HCC development where glycosylation can act as a critical regulatory mechanism^4^. Modification of proteins by glycosylation regulates many biological processes including protein folding, stability, or host–pathogen interactions^5^. For example, glycosylation of the N297 of human IgG is known to modulate interactions with the Fc receptors and subsequent biological and therapeutic responses^6–8^. It is therefore of considerable interest to quantify accurately the IgG glycoforms.

Studies from our and other laboratories have shown that glycosylation of immunoglobulins and various liver secreted proteins provides a means of serologic detection of fibrotic liver disease^9–16^. N-glycosylation of immunoglobulin G (IgG), in particular, has been studied extensively^9,13,14^ but targeted LC–MS/MS quantification of the IgG N297 glycoforms, known to regulate interactions with the Fc receptors and subsequent biological responses has been somewhat limited in the context of liver fibrosis^10,11,17,18^. We have introduced assays for the monitoring of liver fibrosis by quantification of mucin-type O-glycoforms of hemopexin (HPX)^19,20^. The studies jointly suggest that LC–MS quantification of the N- and O- protein glycoforms could serve as a useful tool for the monitoring of progression of the fibrotic liver disease provided that the assays become accurate, fast and reproducible. This would fulfil an urgent need for well-qualified biomarker(s) for non-invasive early detection of HCC.

Here we build on our earlier studies^20^^,^^21^ to introduce a multiplexed microflow LC–MS/MS-PRM (mLC-MS/MS) assay for simultaneous quantification of the N-glycoforms of IgG and sialylated O-glycoforms of HPX. Our optimized method allows complete processing (reduction, alkylation and tryptic digestion of proteins) of the unfractionated serum samples in approximately 3 h using Pressure Cycling Technology (Barocycler NEP2320 EXT, Pressure BioSciences, South Easton, MA)^19^ which is followed by a 5 min analysis of each sample by a targeted LC–MS/MS-PRM assay with online analyte capture, desalting, and gradient elution. We used the method to quantify selected IgG N-glycoforms and HPX O-glycoforms in serum samples of patients with HCV-induced fibrotic liver disease, using a capillary flow LC system (Dionex Ultimate 3000) and Q Exactive-HF mass spectrometer (Thermo). We demonstrate utility of the method in a high-throughput serologic screening setup which opens up the potential for clinically relevant serologic screening of glycopeptide biomarker candidates in the fibrotic liver disease.

## Experimental section

### Materials and reagents

Ammonium bicarbonate, DL-dithiothreitol (DTT), iodoacetamide (IAA) (Sigma-Aldrich, St. Louis, Missouri, USA); mass spec grade Trypsin/Lys-C mix (Promega, Madison, WI, USA). LC/MS grade Water, 0.1% formic acid in Acetonitrile (ACN), 0.1% formic acid in Water (Thermo Fisher Scientific, Waltham, MA, USA). Acclaim PepMap 100 column, PepMap Trap Cartridge (Thermo Fisher Scientific, Waltham, MA, USA).

### Sample processing

Serum samples from control, fibrosis and cirrhosis groups were processed directly by trypsin digestion, without any enrichment step^20^. Briefly, serum samples (2 µl each) were diluted 1:70 with 25 mM ammonium bi-carbonate; and treated with 5 mM DTT at 60^O^C for 1 h, followed by 15 mM iodoacetamide for 20 min at RT in the dark, then 5 mM DTT for 20 min at RT. Proteins in a fixed volume of samples from above (20 µL of the reduced and alkylated diluted serum sample) were digested with mass spectrometry grade Trypsin/Lys-C mix (1 µg) at 37 °C in a Barocycler (60 cycles, 1 min hold at 30kpsi). The peptides were analyzed by LC–MS/MS without any additional processing steps.

### Micro-flow LC–MS/MS-PRM

LC–MS/MS analysis was performed using capillary-flow Ultimate 3000 RSLCnano chromatography system and a Q-Exactive HF Mass Spectrometer (Thermo) with a nanospray source housing a multinozzle M3 emitter spray tip (Newomics, Berkeley, CA, USA)^21^. The samples were loaded onto a PepMap C18 Cartridge (1 mm x 5 mm) and desalted using a sample loading pump at 10 μl/min flow rate 0–1 min with 0.1% formic acid in water, following which the peptides were eluted at a 5 μl/min flowrate. A C18 Acclaim PepMap 100 75 μm × 2 cm nanoViper column was directly connected to the multinozzle emitter. A schematic of the gradient (Supplementary Fig. S1) consists of a 1–2.5 min linear gradient of 1–15% ACN in 0.1% aqueous formic acid, followed by 30 s to 90% ACN, then hold at 90% ACN for 1 min, and equilibration of the column for 1 min at 0% ACN. The valve is switched at the equilibration step, and the trap column is equilibrated at 10 μl/min flow rate making it ready for the next sample injection.

A Parallel Reaction Monitoring (PRM) workflow was used for scheduled MS/MS fragmentation of target ions (isolation window m/z 2.0, HCD fragmentation, resolution 30 K). Table 1 shows the peptide glycoforms analyzed in this study, MS data collection parameters, and transitions used for the quantitation. We analyzed simultaneously selected IgG N-glycoforms of the peptide EEQYNSTYR (G0, G0N, G0FN, G0F, G1, G1N, G1FN, G1F), and mono- and di-sialyated HPX O-glycopeptide TPLPPTSAHGNVAEGETKPDPVTER HexNAc(1)Hex(1)Neu5Ac(1), HexNAc(1)Hex(1)Neu5Ac(2)^11,20,21^. The respective glycan structures are shown in Table 1.

**Table 1:** MS data collection parameters for a targeted PRM analysis, and the transitions used for quantitation of the tryptic glycopeptides of IgG and HPX.

Glycopeptide	Glycan structure	Mass [*m/z*]	CS [*z*]	Polarity	Start (min)	End (min)	NCE	Transitions
G0- EEQYN_297_STYR		830.001	3	+	1	2.5	12	1141.2–1143.2
G0N-EEQYN_297_STYR		897.694	3	+	1	2.5	12	1242.6–1244.6
G0FN-EEQYN_297_STYR		946.38	3	+	1	2.5	12	1317.3–1319.3
G0F-EEQYN_297_STYR		878.687	3	+	1	2.5	12	1215.7–1217.7
G1- EEQYN_297_STYR		884.018	3	+	1	2.5	12	1141.2–1143.2
G1N- EEQYN_297_STYR		951.712	3	+	1	2.5	12	1242.6–1244.6
G1FN- EEQYN_297_STYR		1000.398	3	+	1	2.5	12	1317.3–1319.3
G1F- EEQYN_297_STYR		932.704	3	+	1	2.5	12	1215.7–1217.7
HexNAc(1)Hex(1)Neu5Ac(1) T_24_PLPPTSAHGNVAEGETKPDPVTER		843.6	4	+	2.4	4	20	905.5–907.0
HexNAc(1)Hex(1)Neu5Ac(2) T_24_PLPPTSAHGNVAEGETKPDPVTER		916.4	4	+	2.4	4	20	905.5–907.0
IgG peptide GPSVFPLAPSSK		593.8	2	+	2.6	4	35	417.9–419.9, 699.0–701.0, 846.0–848.0

*HexNAc: blue rectangle (GlcNAc), yellow rectangle (GalNAc).

Hex: green circle (Man), yellow circle (Gal).

Red triangle (Fuc).

Purple diamond (Neu5Ac).

The glycan structure of the peptides is shown.*

### Study population

The sample set was described in our previous study^22^. Briefly, serum samples of participants in the HALT-C trial, whose fibrotic status was determined by ISHAK score based on liver biopsy, were obtained from the central repository at the National Institute of Diabetes and Digestive and Kidney Diseases (NIDDK). The HALT-C trial is a prospective randomized controlled trial of 1050 patients that evaluated the effect of long-term low-dose peginterferon alpha-2a in patients who failed initial anti-HCV therapy with interferon^23^. All the patients in our study were from the control arm of the trial and were compared to disease-free controls frequency matched on age, race and gender; that the controls donated blood samples at Georgetown University (GU) in line with an approved IRB protocol. In this study, we analyzed 15 disease free controls, 15 HCV fibrotic (Ishak score 3–4), and 15 HCV cirrhotic (ISHAK score 5–6) patients. (Supplementary Table S1).

### Data analysis

LC–MS/MS data were processed by Quan Browser (Thermo) to deduce the peak areas based on the listed transition ions (Table 1). The peak areas of the IgG glycoforms were normalized against the log_2_ peak area of an internal IgG peptide GPSVFPLAPSSK, quantified simultaneously. The internal peptide peak area was calculated by summing the intensity of three product ions. For the measure of O-HPX the ratio of disialoT-(HexNAc(1)Hex(1)Neu5Ac(2) over monosialoT-HexNAc(1)Hex(1)Neu5Ac(1) was calculated based on their respective peak areas and defined as S-HPX, as described previously^20^.

Statistical analysis of the datasets was performed using GraphPad Prism software (v9.4.1). The distribution of normalized peak areas of the IgG glycoforms, and S-HPX were compared between the disease groups, and the data was visualized by a nested Tukey plot. The descriptive statistics such as minimum, maximum, mean, and standard deviation were summarized for each analyte. One-way ANOVA tests, two-sample t-tests, and the area under the receiver operating characteristic curve (AuROC) along with sensitivity and specificity were used to evaluate the ability of each analyte to separate the disease groups. In parallel, Kruskal–Wallis and Wilcoxon tests were used to account for non-normality in analytes. A two-sided significance level of 0.05 was used for statistical significance.

### Institutional review board statement

The study was conducted according to the guidelines of the Declaration of Helsinki, and approved by the Institutional Review Board of Georgetown University, IRB code: 2008-549, study: Glycans in Hepatocellular Carcinoma^22^. All participants provided written informed consent.

## Results and discussion

In this study, a multiplexed microflow LC–MS/MS PRM method was developed to quantify simultaneously selected IgG N-glycopeptides and sialylated HPX O-glycopeptides in a 5 min run at a 5 µl/min flow rate. We do this because the fibrotic liver pathology leads to changes in the secreted proteome and its glycosylation which can be efficiently captured by serologic analysis of the glycopeptides. Our prior studies showed that both N-glycosylation and O-glycosylation pathways are altered^20,22,24,25^ and we expected that their simultaneous quantification will provide an improved reflection of the liver pathology. However, fast and robust analytical methods is a prerequisite for a clinically relevant assay which prompted us to optimize this multiplexed fast method.

A standard QE-HF mass spectrometer coupled to Dionex capillary flow LC system proved adequate as the analytical equipment of choice, and the introduction of the analytes to the MS was improved by the use of a multinozzle M3 emitter spray tip (Newomics)^21^. The higher flow rate in mFlow-LC reduces the gradient time in comparison to traditional nanoflow LC–MS/MS, and increases the reproducibility and robustness of the measurements^26,27^. Use of a multinozzle emitter tip that splits the flow evenly into multiple smaller streams enhance the ionization efficiency^28^. This helped us develop a fast, robust method for a multiplex analysis of N- and O-glycopeptides in one analytical run using commonly available equipment. This means that this type of assay can now be adapted for clinical-type sample screening in other laboratories which is one of the goals of our study. Since the IgG and HPX proteins are abundant in serum, the readily available QE-HF mass spectrometer is fully adequate compared to more sensitive mass analyzers on the market or used in our previous studies^11,20,21,25^. To achieve time efficiency, sample preparation from crude serum was performed without any pre-fractionation steps using pressure cycling in a barocycler. This enabled us to complete the sample preparation and MS sample analysis on the same day. The throughput could be further improved by implementing fast reduction, alkylation and sample digestion in one-step using accelerated barocycler techniques^29,30^.

LC–MS analysis was performed using an efficient 5 min microflow gradient. Online analyte captures and desalting was achieved using a trap cartridge at 10 µl min flow rate, followed by analysis using a column connected directly to a multinozzle spray emitter. Sample elution was performed with a 2 min gradient, followed by column regeneration for 1 min, and equilibration for 1 min which is used simultaneously for the next sample loading. This allowed us to complete a sample-run in triplicate in 15 min. This is a highly efficient setup especially in view of the fact that we multiuplex quantification of relevant N- and O-glycopeptides in the same run. Quantification of sialylated O-glycoforms of HPX was performed as it is a potential biomarker; we did not observe changes in sialylated N-glycoforms of IgG in the context of fibrotic liver disease, thus it was not a target in current analysis.

**Figure 1:**
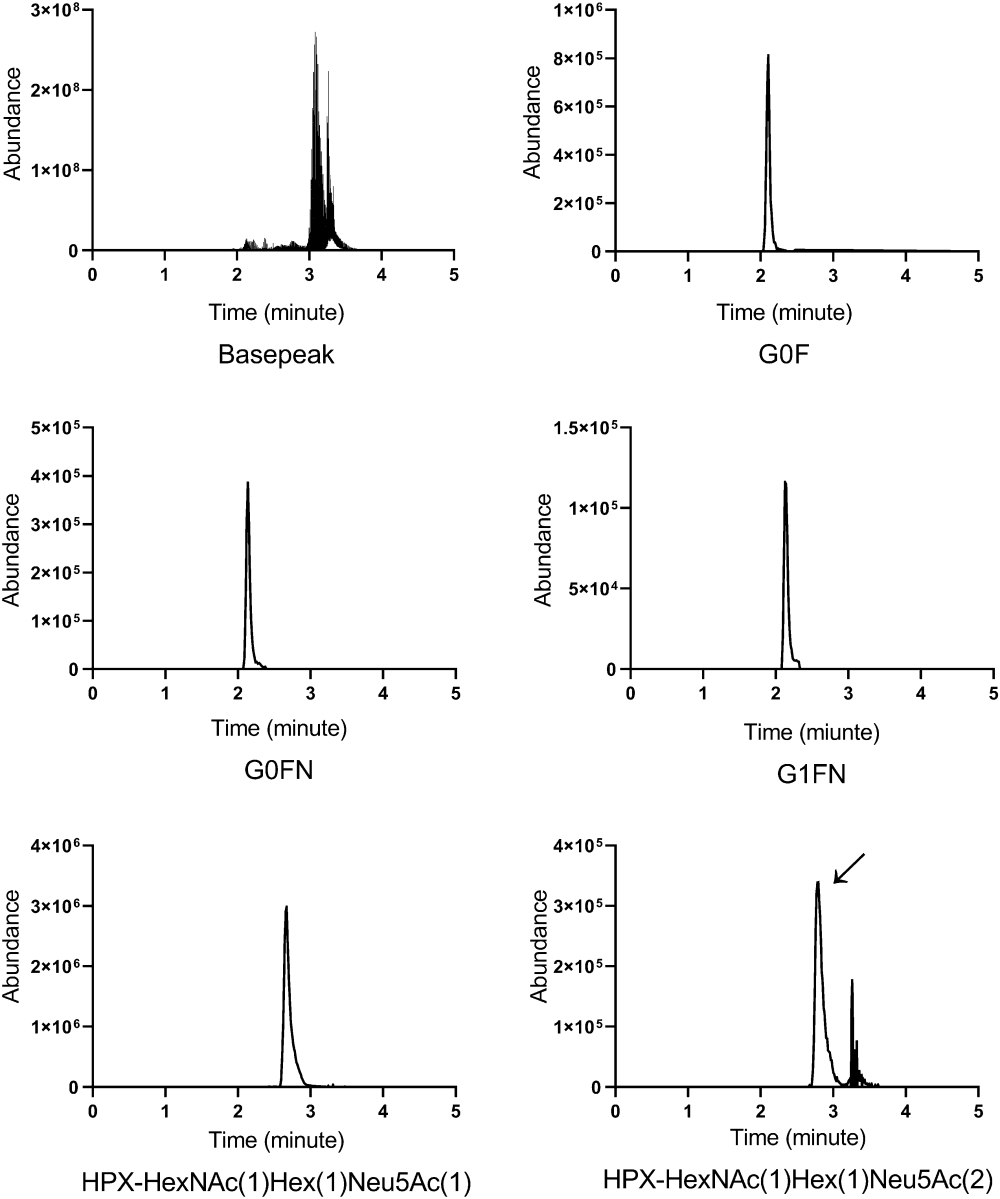
Representative extracted ion chromatograms showing the elution of selected IgG and HPX glycoforms.

We targeted selected glycoforms of IgG N-glycopetide EEQYN_297_STYR, namely G0-HexNAc(4)Hex(3), G0N-HexNAc(5)Hex(3), G0F-HexNAc(4)Hex(3)Fuc(1), G0FN-HexNAc(5)Hex(3)Fuc(1), G1-HexNAc(4)Hex(4), G1F-HexNAc(4)Hex(4)Fuc(1), G1FN-HexNAc(5)Hex(4)Fuc(1), and G1N-HexNAc(5)Hex(4); together with the O-HPX glycoforms HexNAc(1)Hex(1)Neu5Ac(1) and HexNAc(1)Hex(1)Neu5Ac(2) of peptide T_24_PLPPTSAHGNVAEGETKPDPVTER in a multiplexed format (respective glycan structures shown in Table 1). The product ions for the IgG N-glycopeptides were collected at low energy HCD setting^11^. The peak areas of G0, G0N, G0F, and G0FN were calculated from the product ions which resulted from a selective loss of one HexNAc from the parent glycopeptide; the G1, G1F, G1FN, and G1N products resulted from the loss of one HexNAc-Hex fragment. The peak areas of the O-HPX glycoforms were calculated from the loss of the glycan resulting in a peptide backbone transition^20^. The elution profiles of selected informative analytes are shown in Fig. 1.

**Figure 2:**
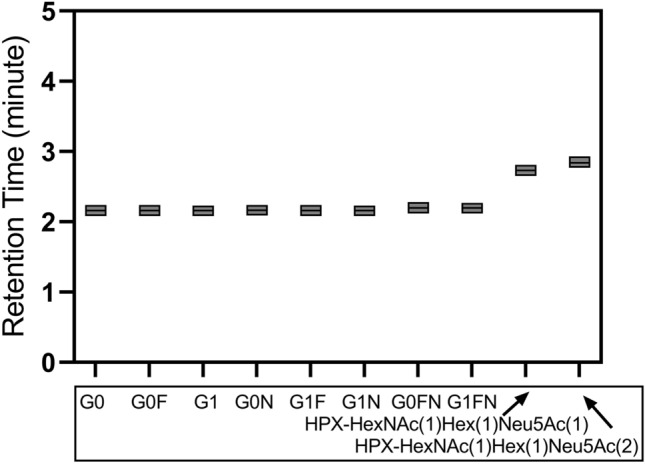
Observed retention times of the IgG and HPX glycopeptides. Each box represents the elution time spread (*y*-axis) of the indicated glycopeptide (*x*-axis), and the mean RT is indicated by a line in the box.

The fast microflow gradient assures that the retention times of the analytes are consistent between the runs (Fig. 2). Peak elution times for the G0 and G1 analytes were 2.16 ± 0.05 min; G0F, G0N, G1F, and G1N 2.16 ± 0.04 min; G0FN and G1FN 2.20 ± 0.04 min; HPX-HexNAc(1)Hex(1)Neu5A(1) 2.73 ± 0.05 min and HPX-HexNAc(1)Hex(1)Neu5A(2) 2.84 ± 0.04 min. Overall, the observed drift of the peak retention time of < 0.1 min over 180 injections demonstrates excellent chromatographic reproducibility.

As a proof of principle, we used the assay to quantify the analytes in the serum of fibrotic (n = 15) and cirrhotic (n = 15) hepatitis C patients compared to a group of disease-free controls (n = 15). The peak areas of the IgG N-glycoforms were normalized to the average peak area of an internal IgG peptide. S-HPX was used as the measure of the O-HPX glycoforms, as described in the methods^20,21^. Statistical analyses were performed to assess the association between the analytes and disease groups and the mean, standard deviation and *p*-values from one-way ANOVA analysis is shown in Table 2. The observed *p*-values for the analytes G0F, G0FN, G1FN, and S-HPX were < 0.001.

**Table 2:** Differences in the analyte distributions in controls and fibrotic or cirrhotic disease groups.

Marker	Mean ± SD			*p* value
	CTRL	FIB	CIR	ANOVA (Kruskal–Wallis)
G0	7955 ± 3670	12,648 ± 4832	16,861 ± 8479	0.001 (0.001)
G0N	41,232 ± 22,263	69,968 ± 31,380	98,070 ± 52,119	0.001 (0.001)
G0F	53,845 ± 23,051	105,243 ± 38,999	133,037 ± 63,708	< 0.001 (< 0.001)
G0FN	19,124 ± 9444	47,675 ± 22,405	57,712 ± 19,918	< 0.001 (< 0.001)
G1	7278 ± 2825	9044 ± 2242	11,782 ± 3796	0.001 (0.003)
G1N	44,160 ± 18,270	49,241 ± 15,714	67,025 ± 29,678	0.018 (0.077)
G1F	72,900 ± 24,774	88,383 ± 21,241	110,607 ± 39,967	0.005 (0.012)
G1FN	10,280 ± 3953	16,698 ± 6450	20,071 ± 3816	< 0.001 (< 0.001)
Mono-sialyated-HPX	150,977,194 ± 42,469,521	106,900,429 ± 39,350,120	78,181,642 ± 34,237,671	< 0.001 (< 0.001)
di-sialyated-HPX	30,787,118 ± 9,482,062	30,177,484 ± 10,252,754	39,805,422 ± 10,405,085	0.020 (0.011)
S-HPX	0.09 ± 0.0296	0.173 ± 0.163	0.349 ± 0.188	< 0.001 (< 0.001)

**Figure 3:**
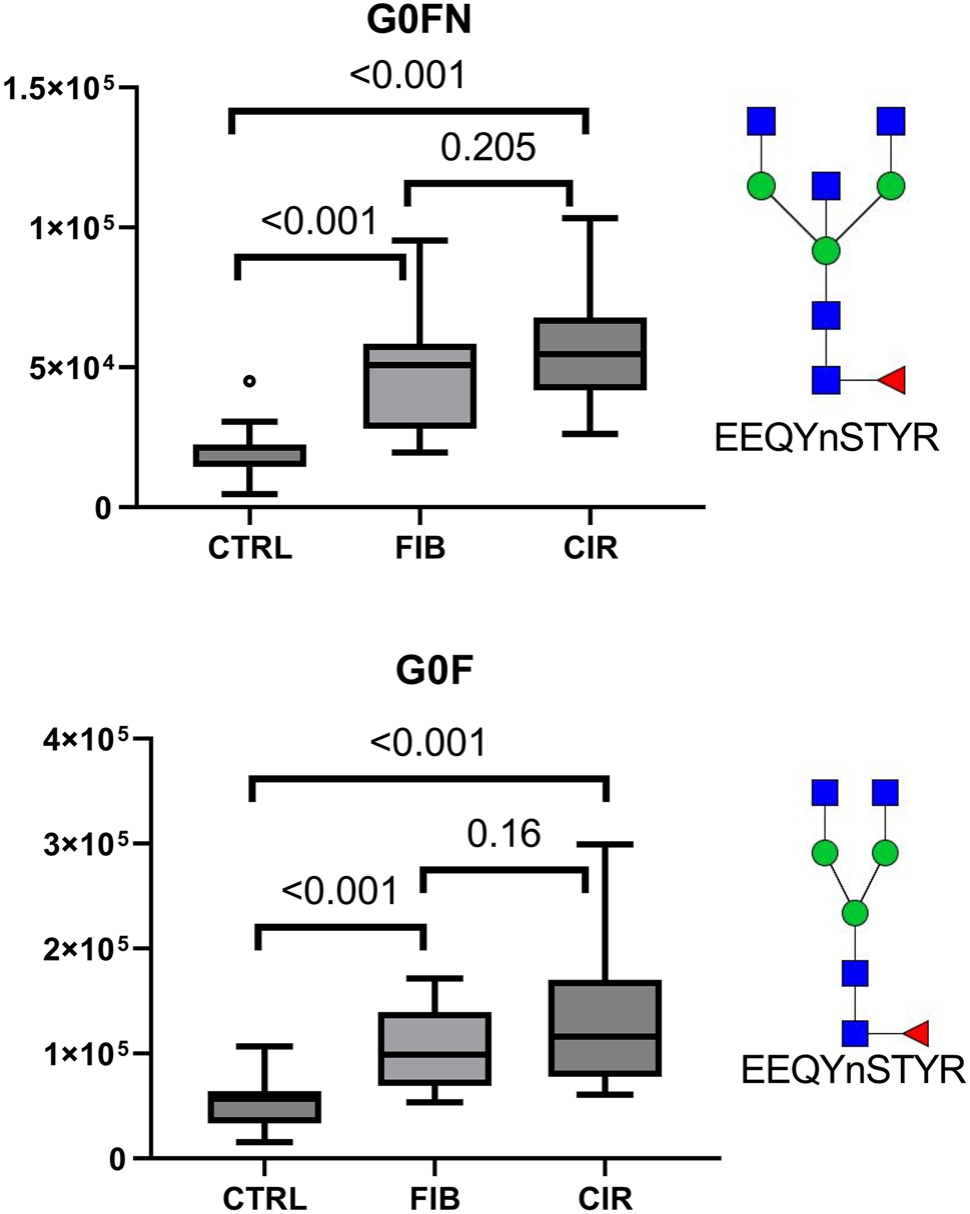
Quantification of the IgG glycoforms in control (CTRL n = 15), fibrosis (FIB n = 15), and cirrhosis (CIR n = 15) patients. IgG1 N-glycoforms G0FN, and G0F increase significantly in the fibrotic patients compared to the control group. The blue rectangle represents HexNAc (GlcNAc), green circle Hex (Man), and red triangle Fuc.

**Figure 4:**
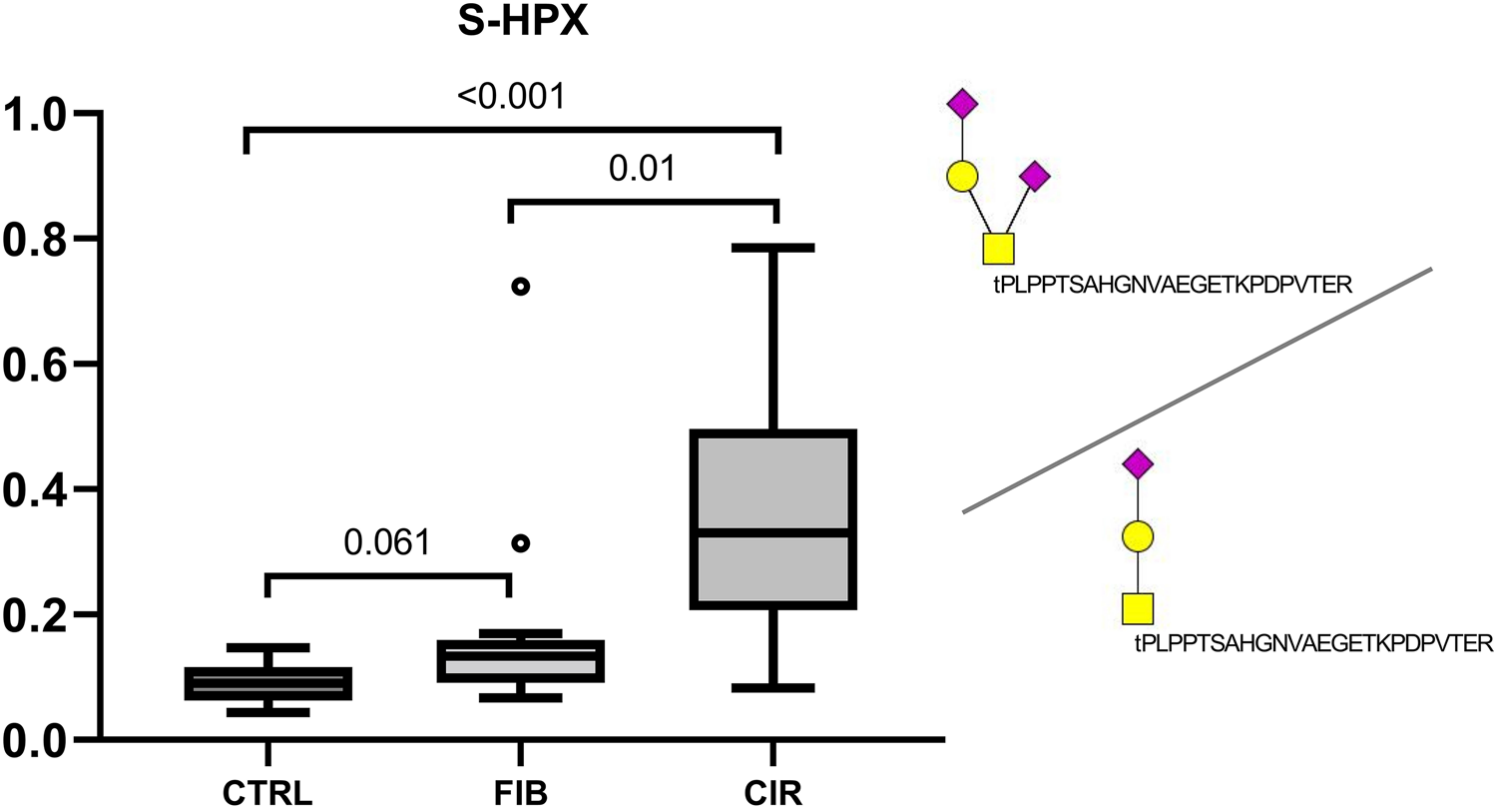
Quantification of S-HPX in the control, fibrosis, and cirrhosis groups (n = 15 each). S-HPX increases significantly in cirrhosis group compared to control (*p* < 0.001) and fibrosis (0.01) groups. The yellow rectangle represents HexNAc (GalNAc), yellow circle Hex (Gal), and purple diamond Neu5Ac.

T-test was performed to assess the association of each analytes between control *vs* fibrosis, fibrosis *vs* cirrhosis, and control *vs* cirrhosis groups (Supplementary Results—Table S2). All of the analytes measured in this study separated control *vs* cirrhosis group (*p* ≤ 0.004, except G1N *p* = 0.017). Most IgG analytes did not separate efficiently fibrotic and cirrhotic patients (*p* > 0.05), except G1 (*p* = 0.02) and G1N (0.05). But they (except G1 and G1N) separate the controls from the fibrotic group. The G0FN (*p* < 0.001) and G0F (*p* < 0.001) glycoforms separate these two groups most efficiently as shown by a nested tukey plot (Fig. 3); please see supplementary figure S2 for the other IgG analytes. To the contrary, the S-HPX separates efficiently the fibrosis and cirrhosis groups (*p* = 0.01) but is less efficient in separating the control and fibrosis groups groups (Fig. 4).

**Figure 5:**
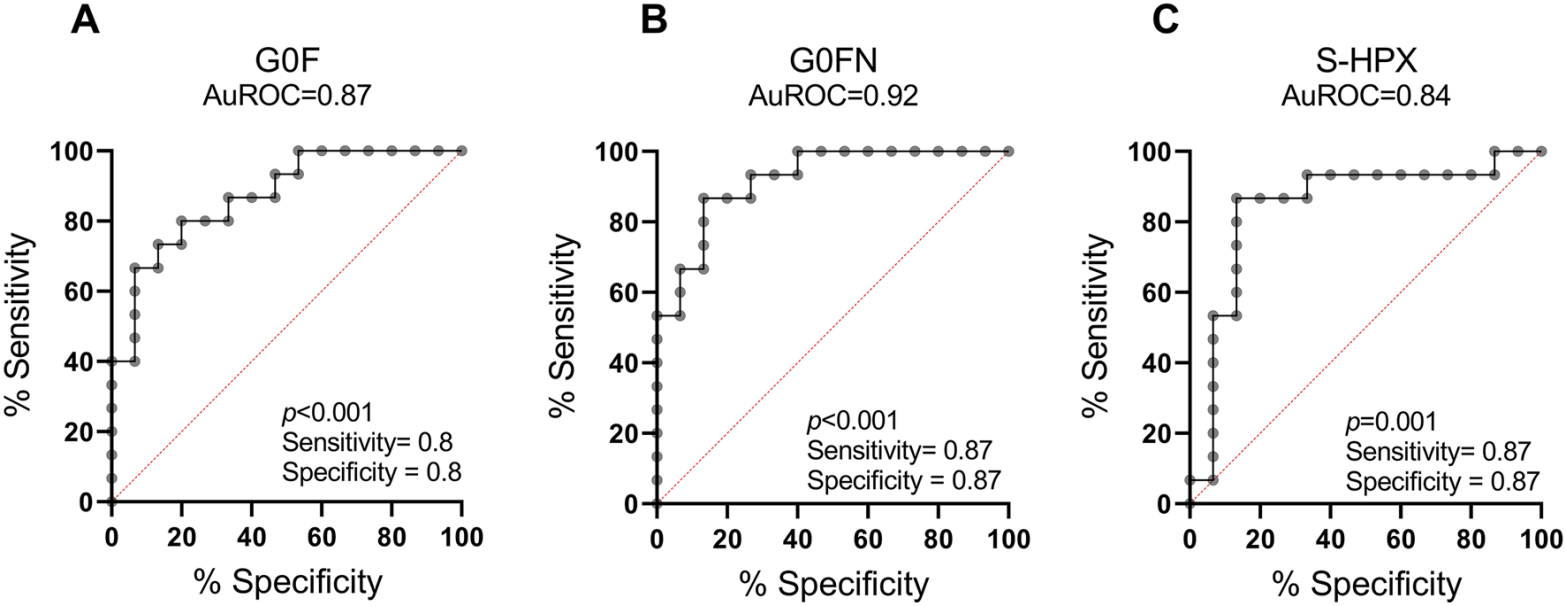
AuROC analyses of the IgG glycoforms G0FN (**A**) and G0F (**B**) between the liver fibrosis and control groups, and of the S-HPX (**C**) between the fibrosis and cirrhosis groups.

The results from above statistical analysis indicate that a combination of the IgG G0FN and G0F with S-HPX may provide an efficient way to assess progression of the fibrotic liver disease from mild to advanced stages. The independent AuROC analyses using the two IgG N-glycoforms to compare normal *vs.* fibrotic groups and S-HPX to compare the fibrotic *vs.* cirrhotic groups confirms that. The G0FN and G0F glycoforms achieve AuROCs of 0.92 and 0.87, respectively, in separating the fibrosis from control groups and the S-HPX separated cirrhosis from control with AuROC of 0.84 (Fig. 5 and Supplementary Table S3).

To summarize, we optimized multiplex analysis of the N-glycoforms of IgG and O-glycoforms of HPX in one analytical run. The simultaneous analysis of the two classes of glycosylated peptides is informative for serologic assessment of liver fibrosis and is completed in 5 min which further reduces time of analysis by 8 min compared to our previous report^21^, a 2.5-fold improvement in time efficiency. We also transferred the analyses to a common LC–MS/MS platform which can be adopted for routine testing. This study measured the N- and O- glycoforms in one LC–MS run which allowed an efficient serologic quantitative assessment of the advancing liver fibrosis by assessment of the two independent glycosylation pathways simultaneously. We propose that the use of the IgG G0FN and G0F in combination with S-HPX can be readily evaluated as a means of non-invasive serologic assessment of liver fibrosis in large clinically relevant datasets. We anticipate that the microflow LC–MS/MS-PRM methods will be a useful avenue for the quantification of various glycopeptides in biological and clinical materials.

## Supplementary Information

Supplementary Information.

## Data Availability

The mass spectrometry data have been deposited to the jPOST repository^31^. The accession numbers are JPST001900 for jPOST and PXD037687 for ProteomeXchange. https://repository.jpostdb.org/preview/8780388286356d3f10eb5c. Access key: 1144.
