# Supplementary material for: A multiplexed microflow LC–MS/MS-PRM assay for serologic quantification of IgG N- and HPX O- glycoforms in liver fibrosis
Source: Sci Rep. 2023 Jan 12;13:606. doi: 10.1038/s41598-023-27382-0 (PMC9837073; doi:10.1038/s41598-023-27382-0)
Supplement: Supplementary file 1 — Supplementary Information. [file 41598_2023_27382_MOESM1_ESM.pdf]

Table S1. Basic information on samples analyzed in this study.							
ID	Disease Group	FEMALE	Age	Race	FIBRO_ISHAK_S00	FIBRO_ISHAK_M24	FIBRO_ISHAK_M48
34916	CIR	0	52	1	6	5	5
35018	CIR	0	51	1	5	5	6
35027	CIR	0	56	1	5	5	6
35036	CIR	0	49	1	5	3	6
35060	CIR	0	58	1	5	3	6
35066	CIR	0	59	1	6	6	5
35074	CIR	0	48	2	6	5	6
35083	CIR	0	47	1	6	6	
35089	CIR	1	54	1	5	6	6
35094	CIR	0	47	2	5	5	5
35150	CIR	0	51	1	6	6	6
35153	CIR	1	55	1	6	5	
35165	CIR	1	53	1	6	6	6
35166	CIR	1	57	2	6	6	6
35181	CIR	0	45	1	6	6	5
				AVG	5.6	5.2	5.69
34926	FIB	0	47	1	3	3	3
34946	FIB	1	40	1	3	3	3
34958	FIB	0	47	1	4	3	3
34967	FIB	1	65	1	3	3	3
34973	FIB	1	53	1	4	4	2
34994	FIB	0	49	1	3	2	2
35008	FIB	0	55	1	4	4	4
35016	FIB	0	41	1	3		4
35033	FIB	0	51	1	4	4	3
35051	FIB	0	55	2	3	4	4
35062	FIB	0	44	1	3	2	2
35077	FIB	0	42	1	4	3	3
35080	FIB	0	47	2	3	1	2
35082	FIB	0	45	1	4	3	3
35098	FIB	1	50	2	3	4	4
				AVG	3.4	3.07	3
Control Group							
14758	CTRL	0	50	1			
13692	CTRL	0	51	1			
15962	CTRL	0	53	1			
20917	CTRL	0	46	1			

20919	CTRL	0	46	1			
13686	CTRL	0	46	1			
16020	CTRL	0	47	1			
13472	CTRL	0	47	1			
20913	CTRL	0	48	1			
20895	CTRL	0	56	2			
20912	CTRL	0	57	2			
20908	CTRL	1	54	1			
16970	CTRL	1	57	1			
14809	CTRL	1	57	1			
20887	CTRL	1	43	2			
			Mean Age	Male Gender	Race Caucasian	Race African American	
	Age	CTRL (n=15)	51	11	12	3	
		Fibrosis (n=15)	49	11	12	3	
		Cirrhosis (n=15)	52	11	12	3	
	Gender	Female	4				
		Male	11				
	Race	White	12				
		Black	3				

Table S2. Statistical results showing probabilistic values of association of analytes and the sample groups

	T-test (Mann Whitney)		
Marker	CTRL vs FIB	CTRL vs CIR	FIB vs CIR
G0	0.006 (0.003)	0.001 (0.001)	0.106 (0.187)
G0F	<0.001 (0.003)	<0.001 (<0.001)	0.161 (0.250)
G1	0.068 (0.148)	0.001 (0.001)	0.023 (0.023)
G0N	0.007 (0.011)	<0.001 (<0.001)	0.084 (0.149)
G1F	0.077 (0.202)	0.004 (0.003)	0.068 (0.074)
G1N	0.421 (0.512)	0.017 (0.030)	0.050 (0.126)
G0FN	<0.001 (<0.001)	<0.001 (<0.001)	0.205 (0.089)
G1FN	0.003 (0.002)	<0.001 (<0.001)	0.092 (0.030)
S-HPX	0.062 (0.007)	<0.001 (<0.001)	0.011 (<0.001)

Table S3. AuROC analysis indicates that the abundance of the G0FN and G0F IgG glycoforms separate efficiently the liver fibrosis and control groups, while S-HPX separate the fibrosis and cirrhosis (advanced fibrosis) groups.

Marker	*AuROC*	*95% Confidence interval*	*p-value*[!]	*Sensitivity*[+]	*Specificity*[+]
Control *vs* Fibrosis					
G0FN	0.92	0.826 - 1.00	< 0.001	0.87	0.87
G0F	0.871	0.747 - 0.996	<0.001	0.8	0.8
G0	0.809	0.652 - 0.966	0.004	0.8	0.73
G1	0.658	0.450 - 0.866	0.141	0.8	0.67
G1F	0.64	0.438 - 0.843	0.191	0.67	0.6
G1N	0.573	0.359 - 0.788	0.494	0.67	0.6
G1FN	0.818	0.668 - 0.967	0.003	0.53	1
G0N	0.769	0.600 - 0.938	0.012	0.47	0.93
Fibrosis *vs* Cirrhosis					
S-HPX	0.844	0.686-1.00	0.001	0.87	0.87

! The p-value is from test of the null hypothesis of AuROC=0.5.
+ Sensitivity and specificity were obtained for achieving the maximum Youden's index (sensitivity+specificity)

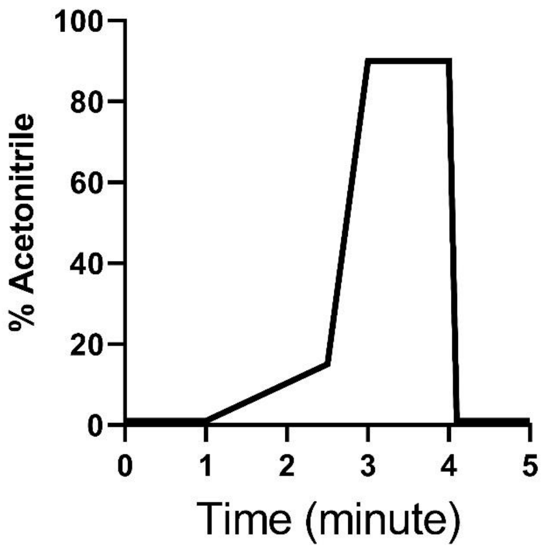

Supplementary Figure S1. Capillary flow gradient profile for LC-MS peptide analysis.

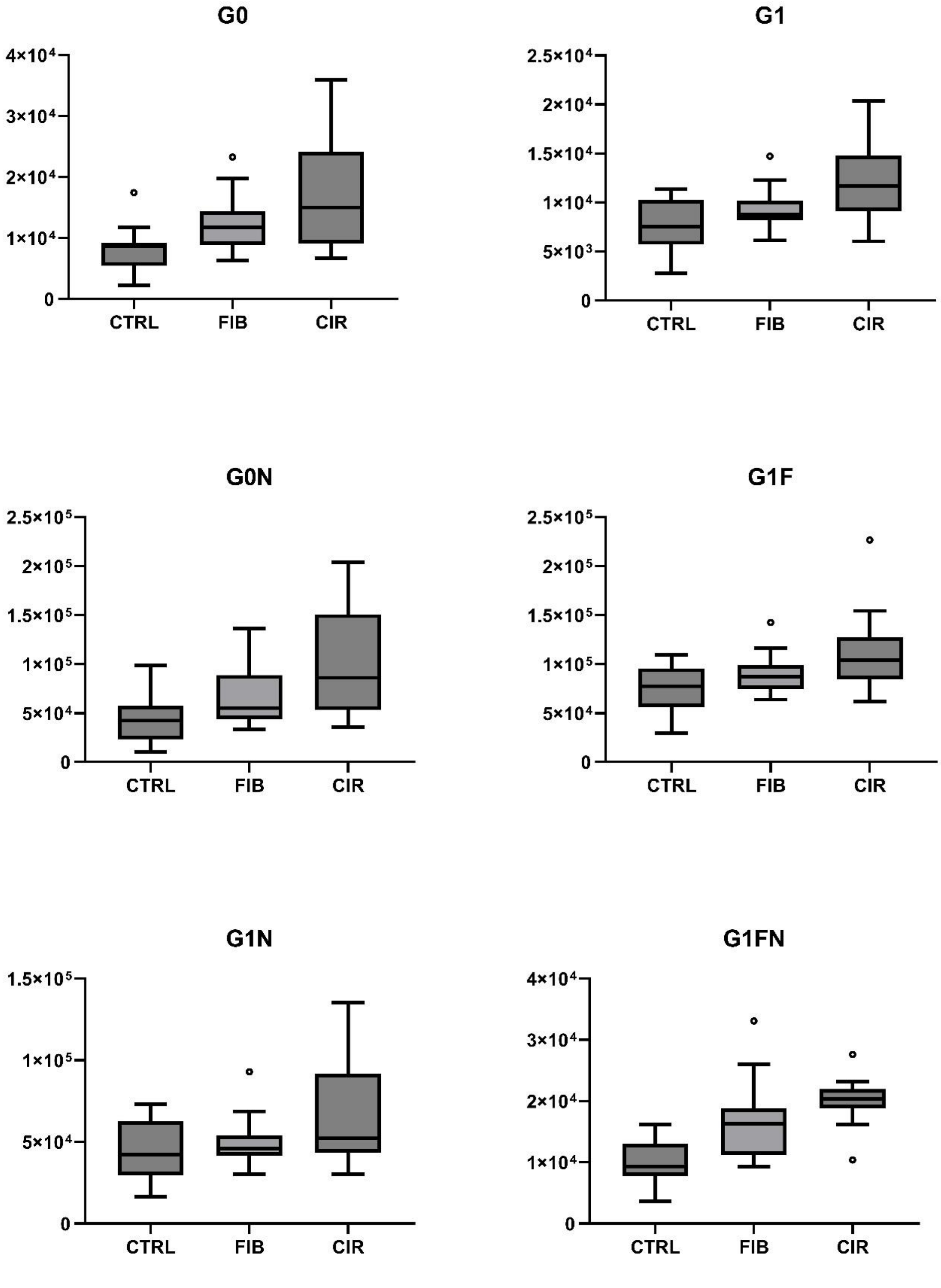

Supplementary Figure S2. Nested tukey plot showing the abundance pattern of indicated IgG glycoforms in control (CTRL n=15), fibrosis (FIB n=15), and cirrhosis (CIR n=15) patients.
